# Preliminary Novel Analysis on Antimicrobial Properties of Concentrated Growth Factor against Bacteria-Induced Oral Diseases

**DOI:** 10.1055/s-0041-1742121

**Published:** 2022-02-21

**Authors:** Muhammad Syafiq Alauddin, Nabilah Mohd Yusof, Aini Sufinah Adnan, Zulfahmi Said

**Affiliations:** 1 Department of Conservative Dentistry and Prosthodontics, Faculty of Dentistry, Universiti Sains Islam Malaysia, Kuala Lumpur, Malaysia; 2 Faculty of Dentistry, Universiti Sains Islam Malaysia, Kuala Lumpur, Malaysia; 3 Department of Basic Sciences and Oral Biology, Faculty of Dentistry, Universiti Sains Islam Malaysia, Kuala Lumpur, Malaysia

**Keywords:** antibiofilm, concentrated growth factor, oral pathogen, platelet-rich concentrate, antibacterial

## Abstract

**Objective** Concentrated growth factor (CGF) is particularly gaining acceptance and popularity in regenerative dentistry. Nonetheless, there are no available studies showing its effect against microorganisms of oral cavity particularly in chronic oral disease-induced biofilms. This *in vitro* research was conducted to determine the antimicrobial effects of CGF against *Staphylococcus aureus* sp. ( *S. aureus* ) and *Streptococcus mutans* sp. ( *S. mutans* ).

**Materials and Methods** Blood samples were obtained from a healthy volunteer. CGF was then prepared using specialized centrifugation equipment (Medifuge, Silfradent, Santa Sofia FC, Italy) and protocol. Antimicrobial activity of the CGF was observed and recorded on standard strains of *S. aureus* and *S. mutans* using a well diffusion method to determine the inhibition zone, broth microdilution to determine minimum inhibitory concentration (MIC) and minimum bactericidal concentration (MBC), and crystal violet assay for biofilm assessment, with chlorhexidine (CHX) 0.12% used as a positive control. Statistical analysis was then performed using one-way analysis of variance followed by Tukey Test post hoc analysis.

**Results** It was observed that there was a presence of clear zones of inhibition around the CGF after 24 hours of incubation. The mean diameter of the inhibition zone was 1.26 ± 0.12 nm and 1.20 ± 0.06 nm for *S. aureus* and *S. mutans* , respectively, with significance difference ( *p* < 0.05) against the control group CHX 0.12%. The MIC values of the CGF against *S. aureus* and *S. mutans* were 47.9% and 34.17%, respectively, and the MBC values of the CGF against *S. aureus* and *S. mutans* were 100%. The viability and the ability in inhibiting the biofilm formation of *S. mutans* and *S. aureus* following treatment with CGF showed a reduction in the concentration-dependent manner as compared with the control group.

**Conclusion** CGF possesses antimicrobial and antibiofilm activity against *S. aureus* and *S. mutans* .

## Introduction

Growth factors are bioactive proteins that control the wound-healing process. One of the initial uses of platelet concentrates in dentistry includes the innovation of fibrin glues used mainly to promote healing by promotion of healing and wounds protection. 1 Platelet concentrates are retrieved from the blood extract and then later developed to promote regeneration of cell by stimulating its growth factor and cell proliferation at the surgical site. Platelets contain enormous volume of key growth factors that include bone morphogenetic protein (BMP), platelet-derived growth factor (PDGF), insulin-like growth factor (IGF), vascular endothelial growth factor (VEGF), transforming growth factor (TGF)-β1, and TGF-β2. 2 3 The first generation of platelet concentrates is platelet-rich plasma (PRP), introduced by Marx in 1988. 2 It was initially developed for medical purposes for its ability in retaining growth factors and promoting better healing by merging the effect of growth factors and properties of fibrin sealant, thus, giving the ideal delivery system of growth factors. 4 PRP has been clinically applied in the procedures of sinus lift and ridge augmentation, socket preservation, alveolar cleft palate repair, and soft tissue procedures such as gingival grafts. 5 The second generation of platelet concentrates consists of platelet-rich fibrin (PRF) and concentrated growth factor (CGF). PRF was developed by Choukroun et al in 2001, produced by centrifuging venous blood, taken without biochemical modification. As a result, a solid fibrin clot is formed that is rich in platelets, leukocytes, and growth factors. 3 Another second-generation platelet concentrate, which is known as concentrated growth factor, developed by Sacco et al, was produced by centrifuging venous blood with its centrifugal technique in a manner similar to PRF, but differed in the specific protocol of centrifugation speed utilizing a specific centrifugation machine. 6 As a result, a harder fibrin structure with richer growth factors was produced compared with the typical platelet concentrates. CGF showed superiority in terms of tensile strength, growth factors, viscosity, and adhesive strength in comparison with the other platelet concentrates. 6 CGF promotes angiogenesis, cell proliferation, matrix remodeling, and regeneration as it contains platelets, leukocytes, CD34+ stem cells, and various growth factors such as PDGF, TGF-β1, epidermal growth factor (EGF), fibroblast growth factor (FGF), IGF-I, as well as VEGF. 6 7 In clinical application, it was proven to be effective in hard tissue regeneration, sinus lift, osteonecrosis, and several other various oral surgery intervention and treatment. 8 9 10 11 12 In a study reported by Pirpir et al, CGF had positive effects on implant stability and accelerated osseointegration. 13

The pathogenicity of oral *Staphylococcus* and *Streptococci* groups remains a pressing challenge for potential intraoral biofilm infection. *Streptococcus mutans* sp. ( *S. mutans* ) is a Gram-positive facultative anaerobe and for decades, it has been widely known as the etiological agents of dental caries. *Streptococci* species are the established early colonizers of salivary pellicle-coated oral surfaces and the most important virulence factor of this species is its biofilm formation ability, which is also known as the dental plaque, with firm adherence to the tooth surfaces. *Staphylococcus aureus* sp. ( *S. aureus* ) on the other hand is a Gram-positive, nonsporadic forming, spherical, and aerobic and facultative anaerobic bacterium belonging to the *Staphylococcus* genus. 14 15 *S. aureus* has been acknowledged as a part of oral flora despite its isolation rates in the oral cavity associated with conditions such as angular cheilitis, suppurative parotitis, denture stomatitis, and acute dentoalveolar infections. 16 In recent studies, it is also found that *S. aureus* may contribute to dental implant failure. 17

There was a plethora of available literature indicating the potential antimicrobial and antibacterial properties of the platelet concentrates especially PRF and its derivatives including i-PRF, H-PRF, and L-PRF. 18 19 20 21 To the best of the authors' knowledge, there is no available literature on antimicrobial properties of CGF against microorganisms to date as no study has been done on this matter. 22 Hence, this study was aimed to investigate the potential antimicrobial properties of CGF on oral *Staphylococcus* ( *S. aureus* ) and *Streptococcus* ( *S. mutans* ) isolates.

## Materials and Methods

### Blood Collection and CGF Preparation

**Fig. 1:**
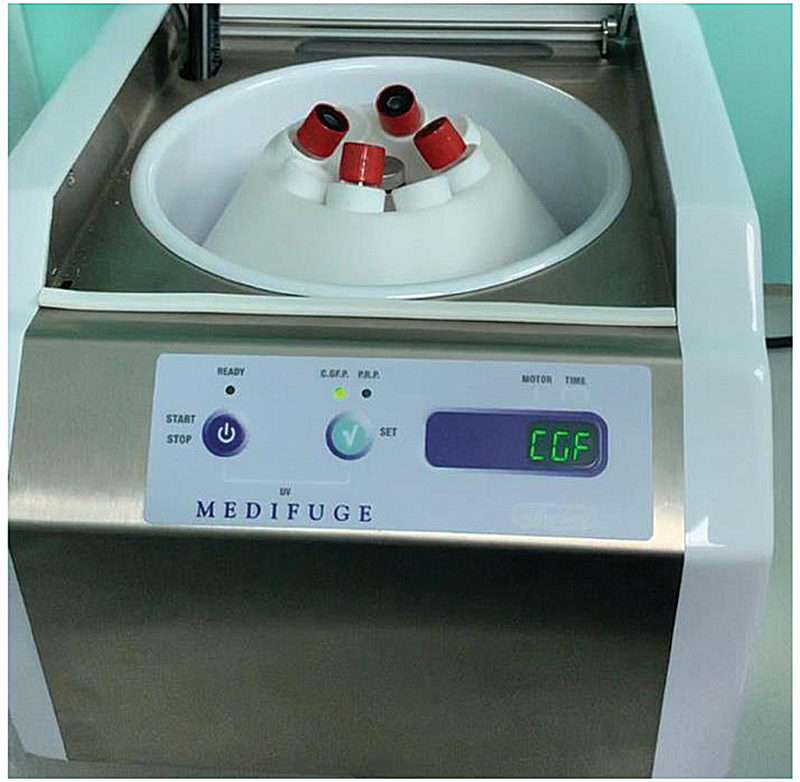
Medifuge MF200 (Silfradent, Italy) with specific program at 30 seconds for acceleration, 2,700 rpm for 2 minutes, 2,400 rpm for 4 minutes, 2,700 rpm for 4 minutes, 3,000 rpm for 3 minutes, and finally with deceleration for 36 seconds.

**Fig. 2:**
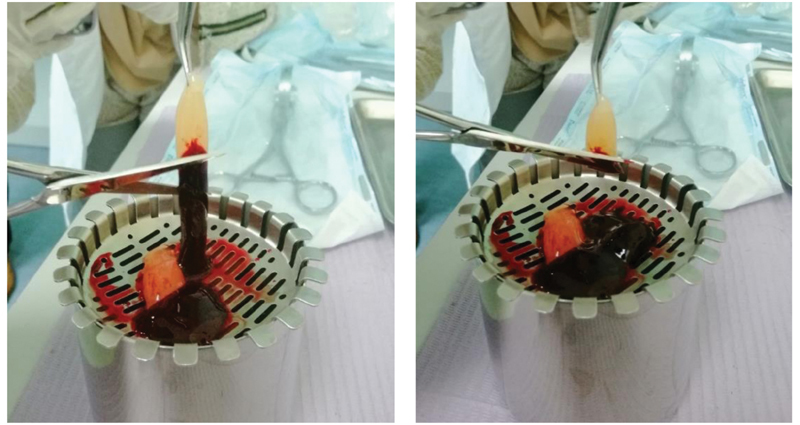
Separation of concentrated growth factor from red blood cell base.

Institution ethical approval was achieved for this study (USIM/JKEP/2020–100). Blood samples were collected from a healthy donor. Verbal and informed information was communicated to the subject followed by the signed consent form. The subject was systemically healthy, a nonsmoker, not pregnant, no history of blood disorder, without symptoms of infection, and was not on antibiotics for the past 3 months prior to this study. A total volume of 20 mL of venous blood was collected by venipuncture in two 10 mL tubes without anticoagulants. The blood samples were centrifuged using a specific program at 30 seconds for acceleration, 2,700 rpm for 2 minutes, 2,400 rpm for 4 minutes, 2,700 rpm for 4 minutes, 3,000 rpm for 3 minutes, and finally with deceleration for 36 seconds using a specialized machine, Medifuge MF200 (Silfradent, Italy; Fig. 1 ). The centrifuged blood resulted in three layers: the uppermost layer of blood serum, the middle layer of fibrin buffy coat, and the bottom layer of red blood cells (RBC). The fibrin buffy CGF clot was removed from the tube using sterile tweezers and then separated from the RBC base using sterile microsurgical scissors as depicted in Fig. 2 . The CGF coat from one tube was compressed and the liquefied CGF (L-CGF) was then collected. The other CGF coat was cut into smaller fragments using the microsurgical scissors to act as the CGF gel (G-CGF). The samples were analyzed immediately prior to the gelation occurrence.

### Microorganisms Preparation

*S. aureus* sp. and *S. mutans* sp. were used in this study. The pure bacterial cultures were then isolated to prepare working cultures. Bacterial isolation in agar plate was performed using the streak plate isolation method by separating the bacteria into four quadrants using a heated inoculating loop. The brain heart infusion (BHI) broth solution was mixed with the pure cultures respectively using a heated inoculating loop. *S. aureus* and *S. mutans* working cultures were then incubated at 37°C for 24 hours.

### Determination of Zone of Inhibition

**Fig. 3:**
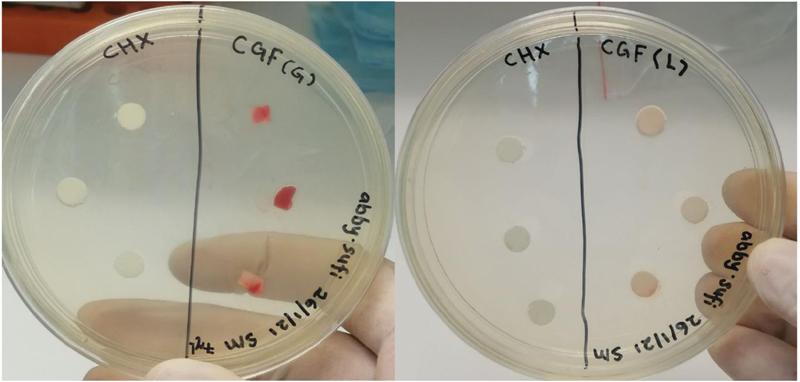
An example depicting inoculation of concentrated growth factor gel and liquefied concentrated growth factor with *Streptococcus mutans* .

The cloudiness of the bacterial suspension in the working broth was then adjusted with a dilution using micropipette in accordance with the 0.5 and 1.0 McFarland's standard solution for *S. aureus* and *S. mutans* respectively. The prepared CGF was screened for its antimicrobial properties using the disk diffusion method. The spread plate method was performed by dipping a sterile cotton swab into the *S. aureus* working broth and then swabbed onto the BHI agar plate. The prepared agar plates were then divided into three sections, G-CGF, L-CGF, and positive control chlorhexidine (CHX) 0.12% solution, and were labeled accordingly as in Fig. 3 . An autoclaved, sterile filter paper disc of 6 mm in diameter was held using a pair of sterile forceps and placed onto the section of the agar accordingly. Then 7 μL of CHX solution was loaded and micropipette onto the paper disc of the CHX section and was repeated for the L-CGF. The G-CGF was placed onto the section using sterile tweezers. The same procedure was repeated for *S. mutans* as well. The plates were then incubated at 37°C for 24 hours. After 24 hours of incubation, the diameter of the zones of inhibition was measured thrice using a ruler and the average measurement was obtained. The growth of the inhibition zone was then measured and the average diameter of the inhibition zone of the three sections for each culture was recorded.

### Minimum Inhibitory Concentration (MIC) and Minimum Bactericidal Concentration (MBC)

**Fig. 4:**
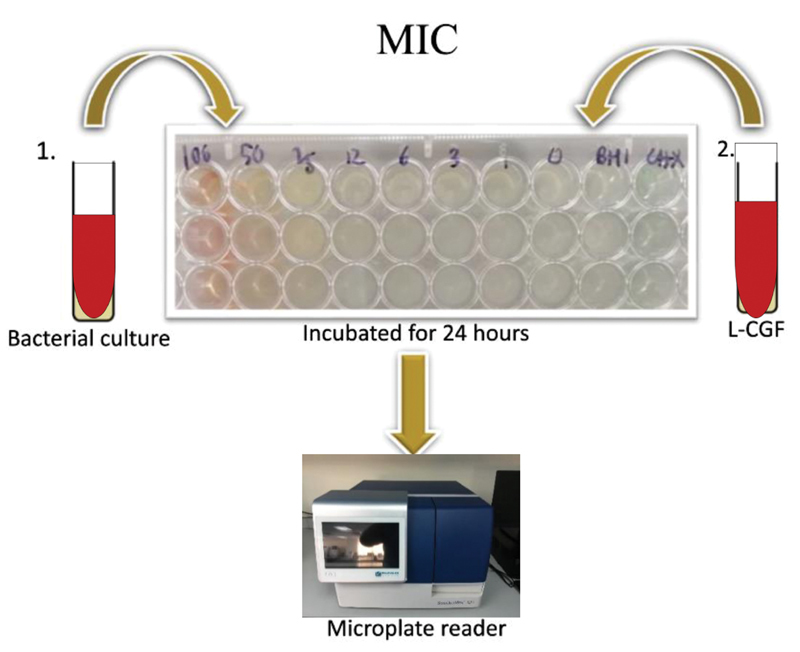
The workflow for Minimum Inhibitory Concentration (MIC) protocol.

To determine the minimum inhibitory concentration (MIC) and minimum bactericidal concentration (MBC), the broth microdilution method was utilized to assess the antibacterial activity of the CGF. An MIC assay was performed onto the 96-well plates using a micropipette to determine the bacterial culture and L-CGF according to its concentration ranging from 100 to 0%. Negative control well was the untreated cell (BHI broth) alone while the positive control well contained cells treated with CHX and the procedure was done in a triplicate manner. After 24 hours of incubation, the plates were placed into the microplate reader to obtain the reading for MIC. The workflow is depicted in Fig. 4 . An MBC test was performed using a micropipette of 5 μL from the MIC wells that exhibited no bacterial growth onto the cultured plates and incubated overnight at 37°C. After 24 hours incubation period, the formation of colonies was recorded.

### Viability Testing

**Fig. 5:**
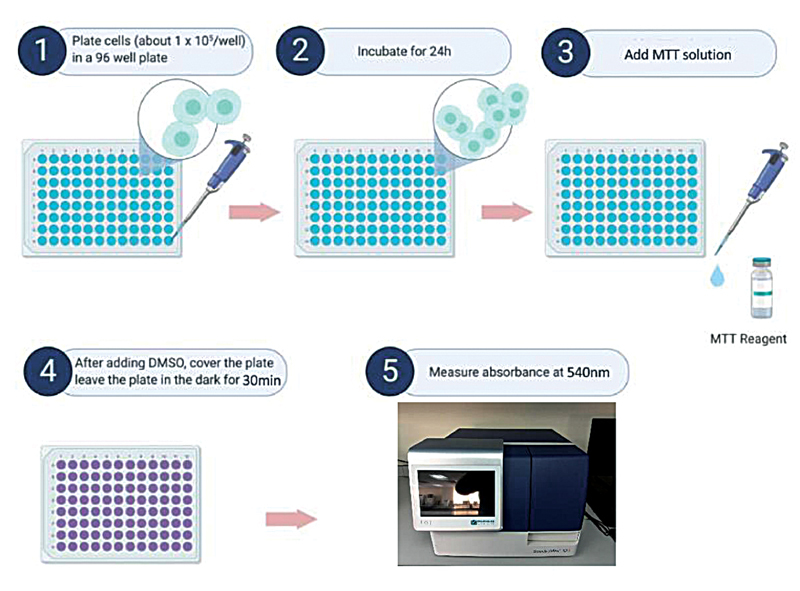
The workflow for cell viability testing protocol.

Viability testing was done to assess the growth pattern of both bacteria following the treatment with CGF. The test was performed by adding 3-(4,5-dimethylthiazol-2-yl)-2,5-diphenyl tetrazolium bromide reagent into the remaining mixture of the 96 wells. The plate was then incubated for 30 minutes. The change in color of the mixture from yellow to purple was observed. The increment in purple resulted from the increasing number of cells recorded. The absorbance was then measured in a microplate reader at 540 nm. The workflow of the viability testing is as per Fig. 5 .

### Biofilm Formation Assay

**Fig. 6:**
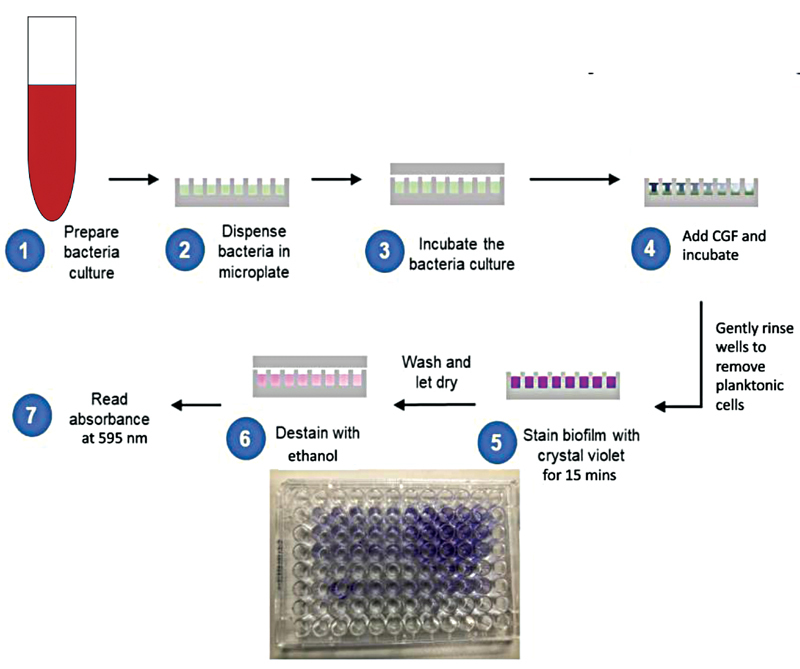
The workflow of biofilm formation assay protocol.

A biofilm formation assay was performed to investigate the effect of the CGF treatment against the biofilm formation. A biofilm formation assay was done by placing diluted bacterial culture into the wells followed with the addition of CGF with a negative control well containing untreated cells and a positive control well containing cells treated with CHX. The plate was then inoculated and incubated, later decanted, and gently washed twice. The adherent bacteria were then stained using a crystal violet dye and then gently washed twice. The bound dye was extracted from the stained cells using ethanol. The biofilm formation was then quantified by measuring the absorbance of the solution at 595 nm in a microplate reader. The workflow of the entire protocol is depicted in Fig. 6 .

### Statistical Analysis

The data obtained from this study were further analyzed using the *Statistical Package for the Social Sciences, version 21.0* software (IBM SPSS; IBM Corporation, New York, United States). A normality distribution was performed initially and further one-way analysis of variance was used. Tukey Test post hoc analysis was performed to analyze which group was the one that gave the significant difference. A *p* -value with less than 0.05 was considered as statistically significant with the sample set at 95% of confidence interval. The data were expressed in mean ± standard deviation.

## Results

### Bacteria Morphological Analysis

**Fig. 7:**
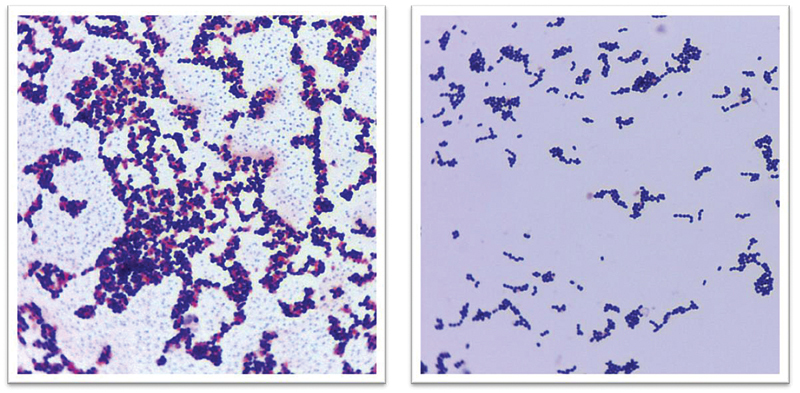
*Streptococcus mutans* (left) and *Staphylococcus aureus* (right) under 100x magnification.

The morphology of *S. aureus* and *S. mutans* was observed under the image analyzer at 100x magnification as per Fig. 7 .

### Determination of Zone of Inhibition

**Fig. 8:**
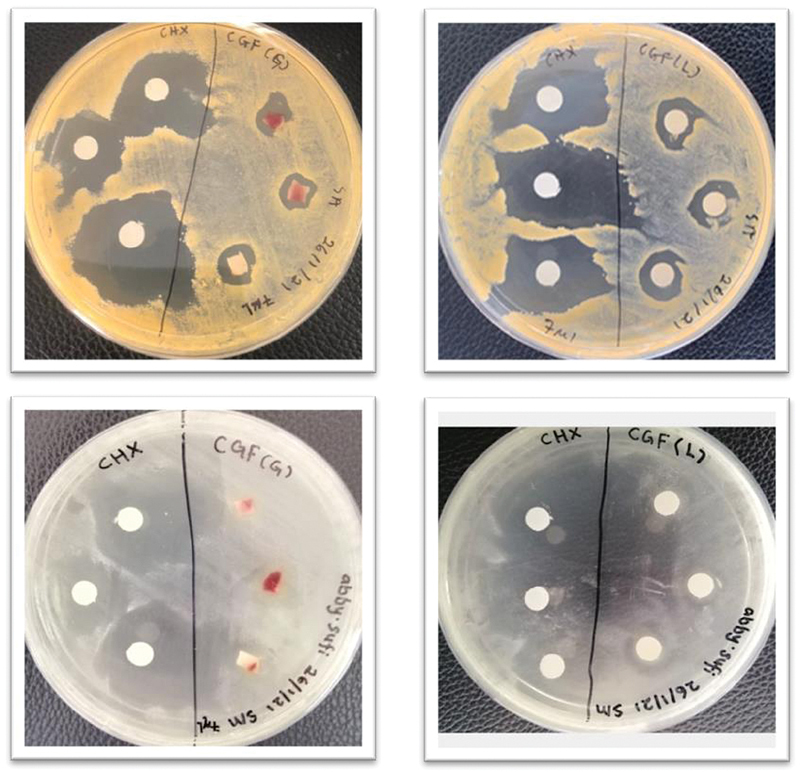
Determination of zone of inhibition showed that both concentrated growth factor gel and liquefied concentrated growth factor produced clear zone of inhibition in comparison to chlorhexidine 0.12% (positive control).

Clear zones of inhibition were observed around CHX, L-CGF, and G-CGF against *S. aureus* and *S. mutans* after 24-hour incubation as per Fig. 8 . The diameter of the clear zones was measured using triplicate readings, and the results are as tabulated in Table 1 .

**Table 1:** Mean values and standard deviation for the diameter of zones of inhibition

Organism	Mean ± SD (cm)		
	G-CGF	L-CGF	CHX (positive control)
*Staphylococcus aureus*	0.92 ± 0.10 *	1.26 ± 0.12 *	2.51 ± 0.32
*Streptococcus mutans*	1.27 ± 0.23 *	1.20 ± 0.06 *	2.83 ± 0.36

Abbreviations: CHX, chlorhexidine (0.12%); CGF-G, concentrated growth factor gel; CGF-L, liquefied concentrated growth factor; SD, standard deviation.

Note: There is significant difference of G-CGF and L-GCF compared with CHX in both *S. aureus* and *S. mutans* .

* *p* < 0.05.

### Determination of Antimicrobial Activity

The antibacterial activity of the CGF was measured using the MIC and MBC assessments. The value of the MIC was measured using a microplate reader with an optical density at 540 nm. The MIC value of the CGF against *S. aureus* and *S. mutans* was 47.85% and 35.59%, respectively. The MBC value of the CGF against *S. aureus* and *S. mutans* was determined at 100%. If the MBC/MIC ratio is ≤4, it was considered as bactericidal while >4 is considered as bacteriostatic. The value of the MBC/MIC ratio of the CGF is 2 and the results are tabulated as showed in Table 2 .

**Table 2:** Determination of antimicrobial activity of concentrated growth factor

Organism	MIC (% v/v)	MBC (% v/v)	MBC/MIC ratio
*Staphylococcus aureus*	47.85 ± 4.75	100	2 (+)
*Streptococcus mutans*	35.59 ± 2.04	100	2 (+)

Abbreviations: MBC, minimum bactericidal concentration; MIC, minimum inhibitory concentration.

### Viability Analysis

**Fig. 9:**
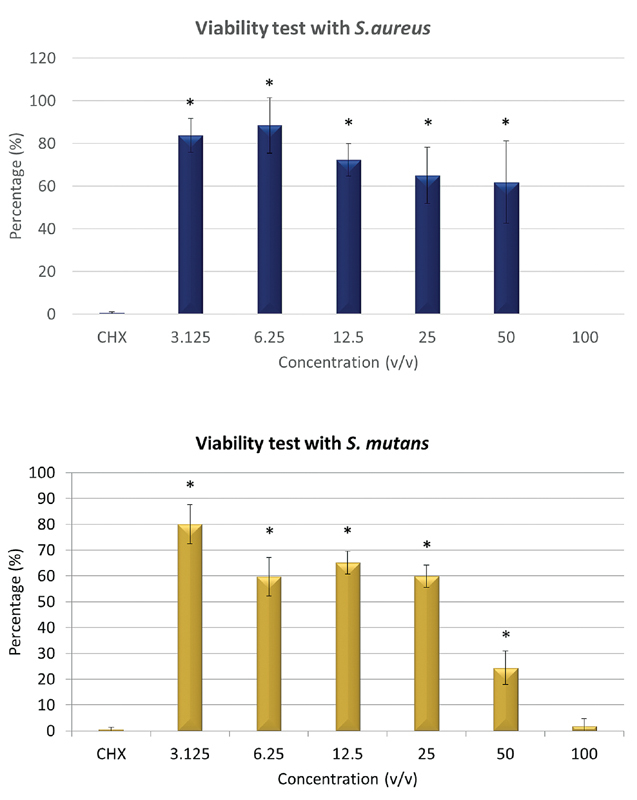
Reduction in overall cell viability of *Streptococcus mutans* and *Staphylococcus aureus* in concentration-dependent manner following treatment with concentrated growth factor with (*) showed significant difference with chlorhexidine 0.12% ( *p* < 0.05).

Inhibition of the *S. mutans* and *S. aureus* growth was observed in a concentration-dependent manner following the treatment with the CGF ( Fig. 9 ). From the results, the CGF at a 100% concentration showed comparable/similar efficacy in inhibiting the growth of both bacteria compared with the CHX ( *p* > 0.05) while other concentrations of the CGF have shown less efficacy in inhibiting both bacteria as compared with the control group ( *p* < 0.05).

### Biofilm Inhibition

**Fig. 10:**
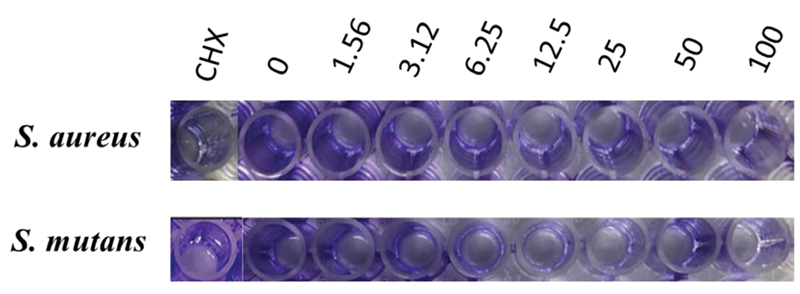
Inhibition of biofilm was observed as the concentration of concentrated growth factor increases.

**Fig. 11:**
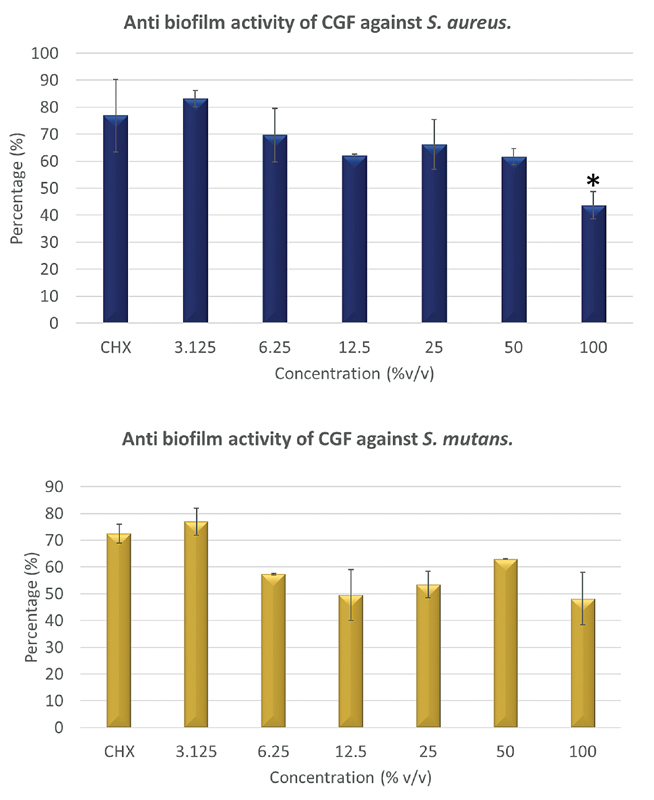
Reduction in capability of concentrated growth factor (CGF) in inhibiting biofilm formation of *Streptococcus mutans and* *Staphylococcus aureus* in concentration-dependent manner following treatment with CGF with (*) showed significant difference with chlorhexidine 0.12% ( *p* < 0.05).

The investigation of the biofilm assay revealed that the inhibition of the biofilm produced by *S. mutans* and *S. aureus* was observed as the concentrations of the CGF increased, as depicted in Fig. 10 . Antibiofilm activity of the CHX against both bacterial strains was also plotted. It showed that the antibiofilm activity of the CGF against *S. aureus* was insignificant ( *p* > 0.05), particularly at 1–50% concentration as compared with the CHX as the positive control. At a 100% higher concentration, the inhibition of the biofilm formation of the CGF in comparison to the CHX ( *p* < 0.05) clearly shows that the CGF is more effective as an antibiofilm agent as compared with the CHX for *S. aureus.* Nonetheless, there was no significant difference ( *p* > 0.05) between the CGF at all concentrations as compared with the CHX against the biofilm produced by *S. mutans* ( Fig. 11 ).

## Discussion

There is a pressing need for the control and inhibition of oral biofilm that is responsible for the formation of dental caries. The pathogenicity of the dental biofilm particularly with the presence and formation of microorganisms within the extracellular matrix will only increase the resistance toward the host immune system and other various antibacterial agents. 23 *S. mutans* is a primary etiologic agent of human dental caries and is particularly effective at forming biofilms on the hard tissues of the human oral cavity. Adherence of *S. mutans* to dental surfaces is the first step in the formation of biofilms by this organism. It was believed to be the primary etiologic agent for the formation and the development of mature dental biofilm that can lead to dental caries. 24 On the other hand, the *Staphylococcus* infections at surgical sites remain one of the widespread postoperative complications. The *Staphylococci* attached to the wound surface is able to proliferate and produce a biofilm. It has been recently recognized that biofilms that produce bacterial infection are prevalent in postoperative wounds and are a causative factor in wound chronicity. 25 26 In addition, *S. aureus* is associated with multiple chronic orofacial and intraoral infections and conditions including facial cellulitis, facial bullous infection, angular cheilitis, dentoalveolar abscess, and dry mouth. 25 26 27 28 The formation of a *Staphylococci* biofilm matrix at the infection site may cause resistance toward treatment by impairing the immune response of the host and may further resist the action of antimicrobial agent. 29 There was also notable prevalence with regard to the resistant strains of *S. aureus* including the methicillin-resistant *S. aureus* that will prolong the hospital care and eventually lead to the impairment of the quality of care to the patients, given their opportunistic and highly communicative nature. 16 30 31 It is paramount to prevent and treat the acquired infection from both microorganisms as early as possible to eliminate and subsequently prevent further advanced chronic oral-related diseases.

Platelet concentrates have been progressively developed due to their regenerative potential and anti-inflammatory properties. In dental procedures, the utilization of platelet concentrates aids in periodontal reconstruction, implant placement, periodontal surgeries, and regeneration. As such, autologous platelet concentrates are widely used and are particularly gaining popularity recently. 7 32 33 Specifically, platelet concentrates such as the PRF is used in treatments of intrabony defects, extraction sockets and recession management, alveolar ridge and sinus floor augmentation, and bone regeneration involving dental implants. 5 The CGF is a platelet concentrate with fibrin network consisting of platelets, leukocytes, and growth factors. One of the main advantages of the CGF is operatively it is easier to be handled and maintained with regard to the desired shape in accordance with the surgical application in comparison to other types of platelet concentrates. 34 This is due to its high cohesiveness that protects it from plasmin degradation, thus giving it a higher fibrin tensile strength and stability. 35 The CGF is similar to the PRF in terms of no bovine thrombin or anticoagulant usage, which in turns reduces the risk associated with the use of additives. 6 Nonetheless, there were differences detected such as when the overall centrifugation protocol and preparation done using different speeds of centrifugation and times, which resulted in a larger, high-density fibrin matrix, richer in growth factors. 6 The usage of the peripheral layer in regenerative procedures is recommended 6 35 due to various growth factors found in the CGF including PDGF, TGF-β, IGF, EGF, FGF, and BMP and supported additionally from the histological perspectives due to specific presence of platelets in the peripheral layer and specific constitution of the fibrin network. Nonetheless, the potential antimicrobial and antibiofilm activities of the CGF are relatively unknown due to lack of evidence in the body of literatures. Therefore, in this study the main aim is to investigate *in vitro* antimicrobial and antibiofilm activities of the CGF and compare it with the conventional 0.12% CHX against two groups of oral pathogenic, Gram-positive facultative anaerobe represented by *S. mutans* and *S. aureus* .

In spite of the widespread interest and preference by the practitioners to use both the gel and liquid forms of platelet concentrate for regenerative and implant therapies, there were extremely limited data in the body of the literature on the utilization and benefits of the liquid form of platelet concentrates specifically for CGF in this study. 36 In the present study, the present forms of the CGF, the G-CGF and L-CGF, were retrieved after the completed centrifugation protocol. The L-CGF was specifically obtained by compressing the CGF membrane using pliers (Silfradent, Sofia, Italy) and the liquid was then collected using a grid and dappen for fibrin separator (Silfradent, Sofia, Italy) accordingly. The results showed that both the G-CGF and L-CGF showed a distinct zone of inhibition on both *S. mutans* and *S. aureus* , where most of the inhibition zone was by the G-CGF on *S. mutans* (1.27 cm ± 0.23) and the minimum amount of inhibition zone was by the G-CGF on *S. aureus* (0.92 cm ± 0.10). The maximum inhibition zone for both microorganisms was recorded by the positive control of CHX 0.12%, which was established as a broad-spectrum antibacterial agent commonly used worldwide as an oral antiseptic mouthwash. 37 This result showed that the CGF in both forms possessed an antibacterial effect on both microorganisms. Additionally, at 100% concentration of the CGF, its effectiveness was at 0.12% of the CHX in inhibiting the growth of both bacteria. Existing literatures showed that the other platelet concentrate forms such as the PRF and i-PRF showed antibacterial effects against a wide array of microorganisms including the *Candida albicans* , *Porphyromonas gingivalis* , and *Aggregatibacter actinomycetemcomitans* by showing positive zone of inhibition with no available reports on the CGF action as an antibacterial agent. 21 38 39 Despite the established data on the antibacterial effect, the exact component within the platelet concentrate and mechanism involved for its antibacterial effect are still largely unknown, and the anecdotal and empirical evidence claiming the production of oxygen ion and metabolites, various antibacterial action including aggregation and binding to the microorganisms, the release of host defense antimicrobial peptides, direct interaction of platelets with microorganisms, specific activation of antioxidants, and production of particular proteins such as defensins, lactoferrins, and cathelicidins that are involved in the host-specific immune response were suggested. 21 39 40 41 42

Bacterial biofilms are serious global health issues and have emerged as key factors in antibiotic resistance due to their abilities to tolerate antibiotics, host defense systems, and other external stresses. Therefore, it contributes to persistent chronic infections. Biofilms not only provide the protection for microorganism from altered pH, osmolarity, nutrient scarcity, and mechanical and shear forces, but also have the ability to block the access of bacterial biofilm communities from antibiotics and host's immune cells. Thus, biofilm matrix provides the additional resistance power to bacteria, which makes them to not only be able to tolerate harsh conditions, but also be resistant to antibiotics. 43 In this study, the results showed that the biofilm formation produced by *S. mutans* was inhibited more effectively after being treated with the CGF as compared with the control group, notably at the highest concentration. However, the effect of the CGF against the biofilm formation produced by *S. aureus* was comparable to the control group at all concentrations. To the best of our knowledge, this study is the first assessment report on the effect of the CGF against the biofilm formation produced by the pathogenic bacteria. This provides an excellent platform for the CGF utilization in a plethora of clinical chronic conditions and orofacial disease especially associated with *S. aureus* infection, such as facial cellulitis, dentoalveolar abscess, and peri-implant infection. 25 26 27 28 44 Since most of clinical findings established in the body of literature focused on application of CGF as a biomaterial in implant-related treatment and maxillofacial and periodontal regenerative therapy, this preliminary finding will enhance the potential synergistic effects of CGF both as a regenerative biomaterial and as a potential antimicrobial agent in those therapies involved. 45 46 47 In implant therapy, various techniques had been advocated to improve the antibacterial effect especially on the surface of the implant and on the implant abutment, namely anodization, nano-modified implant surface, and other methods to maintain a bacteria-free layer on the surface structure and inadvertently aid and enhance the osseointegration process. 48 49 This preliminary finding will further consolidate the utilization of autologous platelet concentrate especially CGF as a natural biomaterial retrieved from the host, with almost negligible biological and biomechanical risk involved, to aid in potential prevention of bacteria growth and domination and thus promote osseointegration. There was another isolated clinical report on utilization of PRP mouth rinse as a treatment modality on nonresponding lichen planus; however, this is merely a single report and warrants more clinical studies. 50 Nevertheless, more consolidated research is necessary for this potential to be translated into an established clinical practice.

## Conclusion

The result of this study demonstrated that the CGF has the potential as an antimicrobial and antibiofilm agent for both *S. aureus* and *S. mutans* isolates. The antimicrobial and antibiofilm capacity of the CGF was comparable to that of the well-established antimicrobial mouth rinse (CHX 0.12%), as reflected by the result. Nonetheless, careful consideration is needed prior to translating this into clinical practice and further in-depth research is needed utilizing *in vivo* and clinical trial models. A more elaborative investigation on the CGF against other major bacteria groups will be beneficial as well.
